# Could Changing the DNA Methylation Landscape Promote the Destruction of Epstein-Barr Virus-Associated Cancers?

**DOI:** 10.3389/fcimb.2021.695093

**Published:** 2021-05-28

**Authors:** Alison J. Sinclair

**Affiliations:** School of Life Sciences, University of Sussex, Brighton, United Kingdom

**Keywords:** Epstein-Barr virus, DNA methylation, CpG motif, decitabine, demethylation, epigenetics

## Abstract

DNA methylation at CpG motifs provides an epigenetic route to regulate gene expression. In general, an inverse correlation between DNA hypermethylation at CpG motifs and gene expression is observed. Epstein Barr-virus (EBV) infects people and the EBV genome resides in the nucleus where either its replication cycle initiates or it enters a long-term latency state where the viral genome becomes hypermethylated at CpG motifs. Viral gene expression shows a largely inverse correlation with DNA hypermethylation. DNA methylation occurs through the action of DNA methyl transferase enzymes: writer DNA methyl transferases add methyl groups to specific regions of unmethylated DNA; maintenance DNA methyl transferases reproduce the pattern of DNA methylation during genome replication. The impact of DNA methylation is achieved through the association of various proteins specifically with methylated DNA and their influence on gene regulation. DNA methylation can be changed through altering DNA methyl transferase activity or through the action of enzymes that further modify methylated CpG motifs. Azacytidine prodrugs that are incorporated into CpG motifs during DNA replication are recognized by DNA methyl transferases and block their function resulting in hypomethylation of DNA. EBV-associated cancers have hypermethylated viral genomes and many carcinomas also have highly hypermethylated cellular genomes. Decitabine, a member of the azacytidine prodrug family, reactivates viral gene expression and promotes the recognition of lymphoma cells by virus-specific cytotoxic T-cells. For EBV-associated cancers, the impact of decitabine on the cellular genome and the prospect of combining decitabine with other therapeutic approaches is currently unknown but exciting.

## Introduction

DNA methylation at the 5-position of the cytosine ring of CpG motifs in DNA provides an epigenetic route to regulate gene expression (Klose and Bird, 2006). Extensive DNA methylation is generally associated with mammalian genes that are not expressed (Suzuki and Bird, 2008), with some cell-type specific genes showing a consistent pattern of DNA methylation found in key regions of the genome (Schmidl et al., 2009).

The methylation of DNA at CpG motifs is achieved and maintained by the interplay of several activities within the cell. 1. *De novo* DNA methyl transferases enzymes (also termed ‘writers’) are responsible for the initial addition of methyl groups to previously unmethylated CpG motifs. 2. Maintenance DNA methyl transferases enzymes are responsible for maintaining the DNA methylation pattern in daughter cells by adding methyl groups to the newly replicated strand of the hemi-methylated CpG motifs that are synthesized during genome replication (Klose and Bird, 2006). 3. A set of ‘eraser’ enzymes work in effective opposition to the DNA methyl transferases, by modifying the methylation mark (Tsiouplis et al., 2020). 4. A set of proteins termed ‘readers’ specifically recognize and bind to methylated CpG motifs and recruit other proteins to that region of the genome. Together, these enzymes act to mark the expression of linked genes (Mahmood and Rabbani, 2019). The mechanism by which the methylated state of the DNA is able to alter gene expression mediate through the action of methyl-DNA binding proteins that recruit chromatin modifiers (Du et al., 2015).

Epstein-Barr virus (EBV) is a common virus that infects people in a generally asymptomatic manner. The virus resides for life within the pool of B-lymphocytes (Thorley-Lawson and Babcock, 1999). For most people, EBV infection is asymptomatic, but it can cause infectious mononucleosis (IM, glandular fever) and a range of cancers both carcinomas (Gastric, Nasopharyngeal) and lymphomas (Burkitt’s lymphoma, Hodgkin’s lymphoma, NK/T-cell lymphoma, diffuse large B-cell lymphoma and post-transplant-like lymphoma) (Vetsika and Callan, 2004; Shannon-Lowe and Rickinson, 2019). EBV lytic replication and production of infectious virus is rare in cancer cells; by this stage the virus has entered into a latent state. The viral genome is maintained in the cancer cells by replicating once per cell cycle in time the host genome, but few viral genes are expressed. The EBV genome consists of double strand DNA and following infection it resides as a circular form in the nucleus of infected cells (Tsurumi et al., 2005; Hammerschmidt and Sugden, 2013). As with the cellular genome, the EBV genome is subject to DNA methylation at CpG motifs and, analogous to the cellular genome, DNA methylation of the viral genome is strongly linked to gene expression (Minarovits, 2006). The DNA methylation pattern of the EBV genome changes during its life-cycle, being largely unmethylated in the infectious virion, then gaining methylation following the infection of cells (Bergbauer et al., 2010). By the time that EBV-associated carcinomas and lymphomas develop, the viral genome is heavily methylated at CpG motifs and few of the approximately 90 viral genes are expressed (Shannon-Lowe and Rickinson, 2019).

The cellular genomes of cancers can also be impacted by DNA methylation. Importantly, some EBV-associated cancers exhibit very high levels of DNA methylation of the cellular genome that correlates with reduced expression of specific tumour suppressor genes (Bass et al., 2014; Stanland and Luftig, 2020).

Deliberately attempting to reduce the DNA methylation in EBV-associated cancer with the aim of activating the expression of cellular and viral genes has the potential to achieve two potentially therapeutic events. First, it may result in reactivation of the expression of viral genes that expose the cells to attack by the immune system. Secondly, it may result in the expression important cellular genes that cause the cancer cells to stop proliferating or to die through apoptosis. Deliberately attempting to alter DNA methylation therefore presents an attractive research direction which may lead to new therapeutic approaches to treat EBV-associated cancer in the future.

### Enzymes That Regulate DNA Methylation

**Figure 1:**
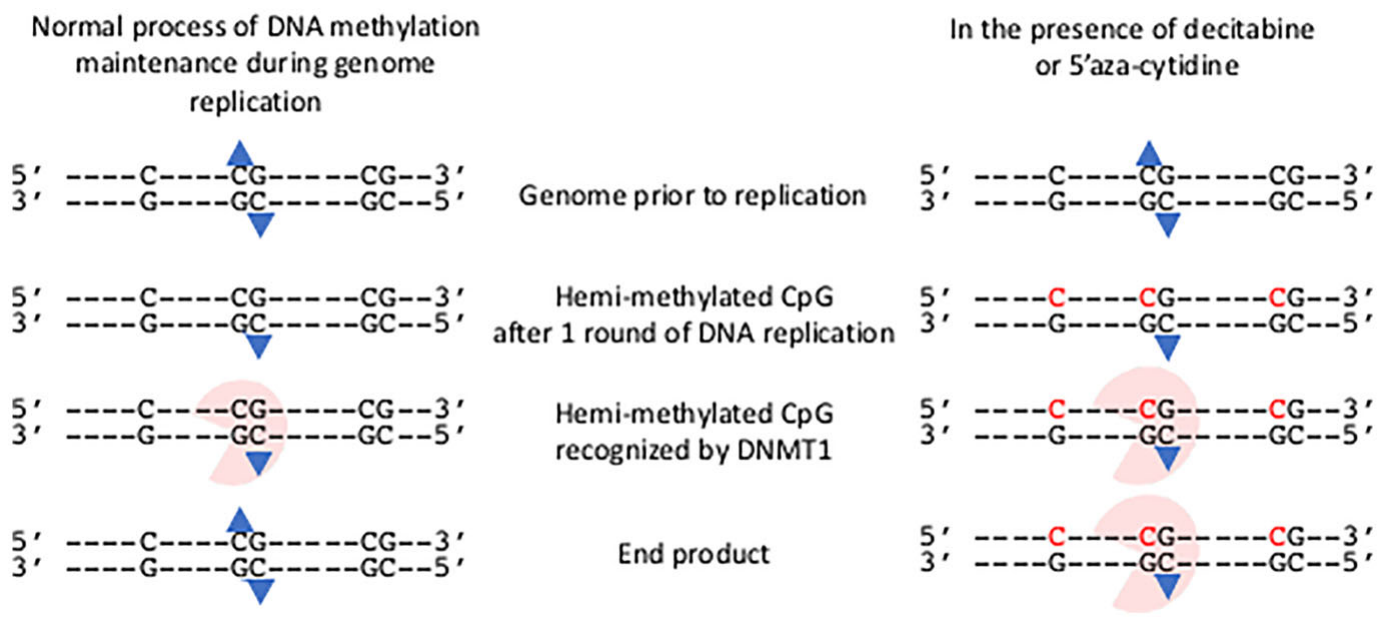
Impact of decitabine and 5’ azacytidine on the maintenance methylation of DNA during genome replication. The presence of an isolated C and two CpG motifs in genome are shown prior to replication. The methylation group on each methyl cytosine of the central CpG motif is shown (blue triangle). Following one round of semi-conservative DNA replication hemi-methylated CpG motifs occur. These are recognized by DNMT1 and a methyl group added to the unmethylated cytosine. Thus the end product has the same methylation state as the genome prior to replication. In the presence of decitabine or 5’aza-cytidine, these modified bases are incorporated into the newly synthesized strand (shown in red). The hemi-methylated CpG motifs are recognized by DNMT1, and are covalently bound to the modified cytosine bases. The genome remains in a hemi-methylated state.

The process of establishing this epigenetic change to gene expression is undertaken through the deposition of DNA methylation onto the cytosine residues of CpG motifs in genomes by *de novo* DNA methyltransferase (DNMT) enzymes that add a methyl group to the cytosine of specific CpG motifs (Klose and Bird, 2006). The individual roles of *de novo* DNA methyl transferases DNMT3A and DNMT3B for methylating regulatory regions of developmental genes and the X-chromosome respectively, have been established and they are also candidates to accomplish *de novo* DNA methylation in somatic cells (Law and Jacobsen, 2010). Once established, the DNA methylation pattern is copied during each cycle of DNA replication by the action of the maintenance DNA methyl transferase, DNMT1 (Law and Jacobsen, 2010); thereby maintaining the set pattern of DNA methylation in daughter cells. As methylated DNA replicates, hemi-methylated CpG motifs consisting of the methylated template DNA strand and the newly replicated and so unmethylated strand are produced. DNMT1 recognizes the hemi-methylated CpG motif and adds a methyl group to the cytosine (**Figure 1**).

The effective removal of CpG DNA methylation on the host genome occurs through one of two routes. If the maintenance DNMT enzyme is compromised during DNA replication then hemi-methylated CpG motifs will be present after one round of replication with the potential for fully unmethylated CpG motifs after a second round of replication. The second route is achieved through the actions of a set of proteins (Ten-Eleven-Translocation (TET) family) that further modify the methyl-group on CpG motifs. (Tsiouplis et al., 2020).

### DNA Methylation of the Host and Viral Genomes in EBV-Associated Cancer

Global methylome analysis of over 30 different cancer types by The Cancer Genome Atlas Network (TCGA) revealed that the highest level of hypermethylation of cellular DNA occurred in Epstein-Barr virus associated Gastric Carcinoma (Bass et al., 2014). Affected genes include a set of tumour suppressor genes (Okada et al., 2013; He et al., 2015; Nishikawa et al., 2018). Hypermethylation of cellular DNA is also seen in Epstein-Barr virus associated Burkitt’s lymphoma and Nasopharyngeal carcinoma, but to a lesser degree (Stanland and Luftig, 2020).

In EBV-associated carcinomas and lymphomas, the EBV genome is also subject to widespread DNA methylation and highly restricted patterns of viral gene expression (Minarovits, 2006). Immediately following infection of cells, transient low-level expression of many of the 90-viral genes occurs from the unmethylated EBV genome. However, the viral genome is rapidly subject to DNA methylation at CpG motifs and settles into one of several highly restricted sets of viral latency gene expression (Tierney et al., 2000; Bergbauer et al., 2010). Three major patterns are common in cancer (latency I, II (also termed IIa) and III), but other patterns of viral gene expression exist including Wp-restricted (Kelly et al., 2009) and IIb latency (Price and Luftig, 2015). Around 90% of the EBV genes are not expressed in any lymphomas and carcinomas, and for the majority of the EBV-associated cancers, the promoters driving expression of the genes generating the most immunogenic T-cell responses (EBNA 2 and 3 family) are not expressed (Minarovits, 2006). In addition, the lytic replication cycle genes (especially BZLF1, BRLF1, BMRF1 and BMLF1) which are highly immunogenic are also silent (Taylor et al., 2015). So, the EBV genome itself provides a wealth of immunogenic genes that are often silenced by DNA hypermethylation.

Clues about how the hypermethylation of cellular and viral DNA occurs in EBV-associated cancer comes from cell culture experiments where it was demonstrated that *de novo* methylation of cellular DNA occurs within days of EBV infection (Queen et al., 2013). Two EBV genes have been implicated: LMP1 and LMP2A. LMP1 activates the expression of both *de novo* and maintenance DNA methyl transferases (Tsai et al., 2006; Peng et al., 2016) and so could both aid the initiation and ensure the maintenance of the DNA methylation changes. LMP1 is expressed in some but not all EBV-associated cancer, however, it has been shown to be expressed transiently after EBV infection of gastric epithelial cells (Matsusaka et al., 2017) in addition to during the immortalization of B-lymphocytes by EBV. It is therefore conceivable that LMP1 might play a transient early role in reprogramming DNA methylation in EBV-associated cancers. In addition, LMP2A which is expressed in some but not all, EBV-associated cancers, drives the upregulation of the maintenance DNA methyl transferase (Hino et al., 2009) and the down regulation of TET gene expression (Namba-Fukuyo et al., 2016). These changes indicate that LMP2A is also a candidate to contribute to global increases in DNA methylation in EBV-associated cancers.

A further piece in this puzzle is the enduring impact of EBV on hypermethylation of the cellular genome even after the viral genome has been lost from the cells (Birdwell et al., 2014). So, clues about the mechanisms by which the hypermethylation of the cellular and viral genomes occurs have been identified and it is possible that LMP1 and LMP2A play transient roles in reprogramming DNA methylation early after entry into cells.

The correlation of EBV-associated gastric cancer with DNA hypermethylation of the cellular genome, the potential contribution of EBV genes to changing the expression of components of the DNA methylation machinery and the relevance of the genes that are potentially silenced by hypermethylation in EBV-associated gastric cancer all support the hypothesis that actively demethylating the genome of EBV-associated cancer cells may be beneficial.

### Impact of Changing the DNA Methylation in EBV-Infected Cells

Attempts to alter the DNA methylation within EBV-infected cells focus on the maintenance DNA methyltransferase. The impact of totally inhibiting the action of the maintenance DNA methyl transferase would be the hypomethylation of the cellular and viral genomes within 1-2 rounds of cell replication (Mani and Herceg, 2010). The reduction in the DNA methylation landscape resulting from this in EBV infected cells is predicted to have impacts on the expression of both viral and cellular genes with the potential for immunogenic viral genes rendering the cancer cells susceptible to attack by the immune system and of tumour suppressor genes reducing growth and survival of the cancer cell. However, global demethylation is a not a targeted approach and it may also drive unfavorable changes in cellular gene expression.

Cytidine analogues that irreversibly inhibit the maintenance DNA methyl transferases and so promote hypomethylation of DNA were identified over 50-years ago (Sorm et al., 1964; Sorm and Vesely, 1968) - 5’Azacytidine (4-Amino-1-(β-D-ribofuranosyl)-1,3,5-triazin-2(1*H*)-one; azacytidine, 5-aza-CR; Vidaza^®^) and its deoxyribose version, Decitabine (2′-Deoxy-5-azacytidine, 4-Amino-1-(2-deoxy-β-D-ribofuranosyl)-1,3,5-triazin-2(1H)-one, 5-aza-CdR; Dacogen^®^). Both cytidine analogues are processed within cells to the triphosphate derivatives and can be incorporated into newly synthesized DNA during the S-phase of the cell replication cycle (**Figure 1**) (Mani and Herceg, 2010). Once embedded within the genome those modified bases form hemi-methylated CpG motifs that are recognized by the maintenance DNA methyl transferase and they irreversibly trap and inhibit the DNMT1, leading to rapid hypomethylation of CpG motifs throughout the genome (**Figure 1**) (Howell et al., 2010; Mani and Herceg, 2010).

Preclinical studies showed that both 5’azacytidine and decitabine are highly active cytotoxic agents against many types of cancer cells (Howell et al., 2010). Furthermore, clinical studies showed that both 5’azacytidine and decitabine are beneficial for people with some forms of haematological cancer (Silverman et al., 2002; Kantarjian et al., 2006; Fenaux et al., 2009; Benton et al., 2014; Mayer et al., 2014). A 5’Azacytidine small scale trial in patients with EBV-associated cancers revealed that demethylation of the viral genome was successfully achieved (Chan et al., 2004).

### Impact of Decitabine as a Demethylation Agent of the EBV Genome in EBV-Infected Cells

**Figure 2:**
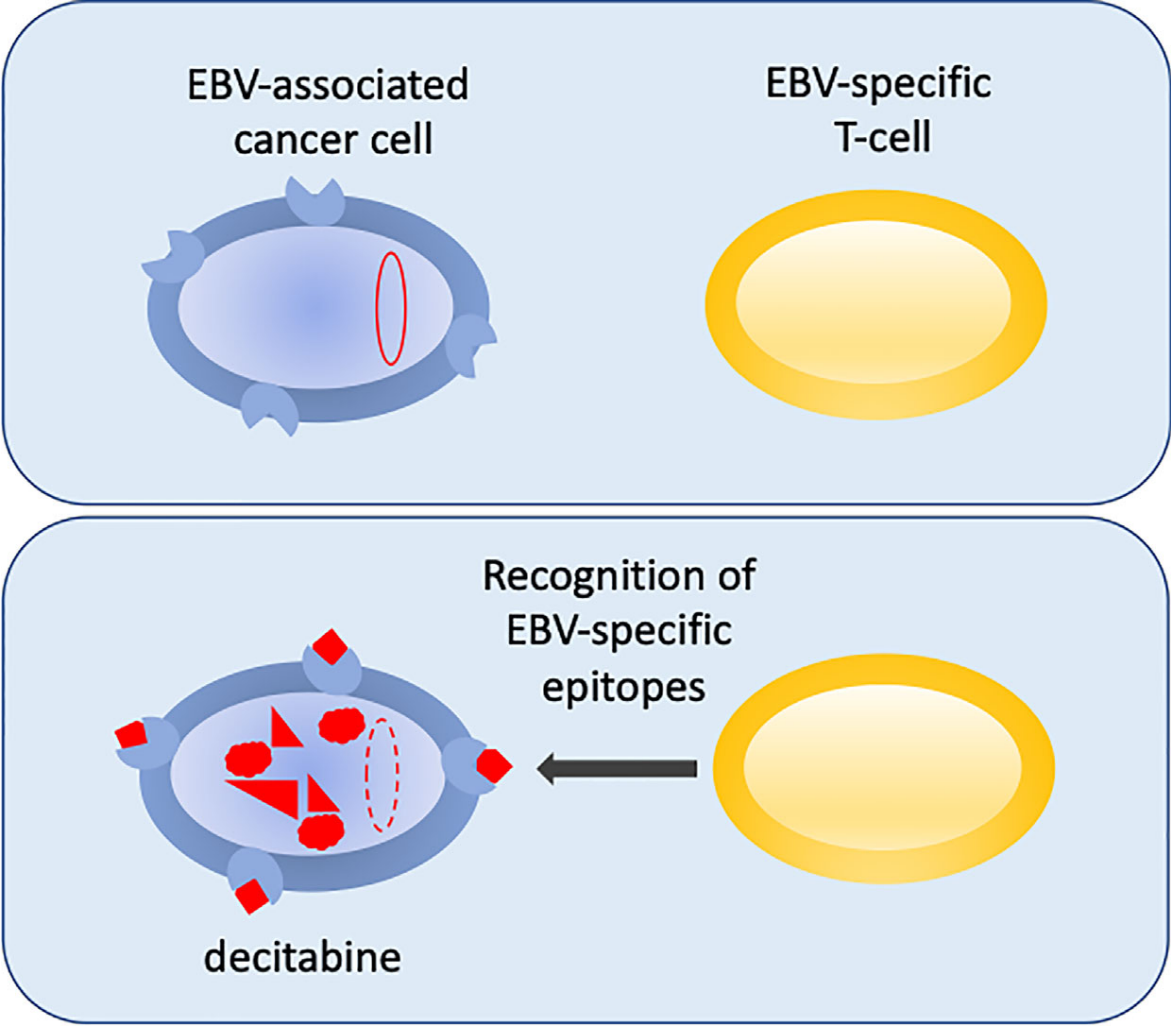
Reversing epigenetic silencing of the viral genome allows recognition by T-cells. EBV-associated cancer cells are shown in blue. In the top panel the viral genome (red oval) is hyper methylated at CpG motifs and largely silent. MHC is shown at the cell surface (blue shape). In the bottom panel, following decitabine treatment, the genome is de-methylated (red dashed-line oval), reactivated and expresses immunogenic viral proteins (red) that are displayed with MHC as peptides at the surface of the cell. This allows the EBV-specific T-cells (yellow) to recognize the cancer cells.

An exciting new development in the fight to reprogram the epigenetics of EBV-infected cells to make them sensitized to immunotherapy was reported recently (Dalton et al., 2020). This builds on previous successes of infusing virus-antigen-specific autologous or allogeneic CTLs to treat EBV cancers (Heslop et al., 2010; Bollard et al., 2014; Bollard and Barrett, 2014; Chia et al., 2014; Kazi et al., 2019; Prockop et al., 2020). Dalton hypothesized that if they could reactivate expression of viral antigens using sub-cytopathic doses of drugs, the cancer cells may become primed for immune attack. In a search to re-purpose drugs that could promote the expression of antigenic viral genes in the aggressive B-lymphomas that express the most restricted pattern of EBV gene expression (latency I), they identified decitabine as a strong candidate to reprogram the expression of immunogenic viral latency genes. Decitabine induced the expression of the two key viral promoters, with the resulting proteins identified in 27-58% of cells in a cell culture model system (**Figure 2**) (Dalton et al., 2020). Excitingly, the reprograming of viral gene expression was reproduced in a more physiologically challenging mouse xenograft tumour model and the change in gene expression was shown to have a durable impact. Importantly, EBV-specific CTLs were able to recognise and kill the decitabine-treated tumour cells *in vitro* and critically they were shown to infiltrate the xenograft tumours *in vivo* (**Figure 1**) (Dalton et al., 2020), providing proof-of-principle.

## Discussion

The pressing question is whether decitabine treatment could provide a relevant clinical approach to treat EBV-associated lymphomas and carcinomas? Decitabine has a proven potential to drive hypomethylation of the viral genome and to prime EBV-associated lymphoma cells to re-express immunogenic viral genes (Dalton et al., 2020). This provides the first part of a potential therapeutic strategy. In combination with the action of the patient’s existing immune response, this may be sufficient to boost an immune attack, or this could be supplemented by infusion of EBV-specific CTLs or T-cells expressing EBV-specific chimeric antigen receptors (CAR-T therapy) (Chicaybam et al., 2019; Dragon et al., 2020; Prockop et al., 2020; Heslop et al., 2021). However, a potential limitation to this approach is that the EBV genome was not reactivated in every lymphoma cell following decitabine treatment (Dalton et al., 2020), and so not all tumor cells would be rendered susceptible to immune attack. Whether reactivation could be stimulated to generate a homogeneous response remains an open question. Interestingly, the sub-cytopathic dose of decitabine used appeared sufficient to stop the lymphoma growing (Dalton et al., 2020). Alterations to host gene expression might also occur as a result of decitabine and may negatively impact on the growth or survival of tumor cells. The recent discovery that LMP1 directs the expression of a set of tumor-associated antigens in a B-lymphoma that are recognized by T-cells (Choi et al., 2021) supports this avenue, although it is not known yet whether the changes in gene expression involves a change in DNA methylation. Changes in expression of cellular genes may be especially relevant for EBV-associated carcinomas, where a high proportion of the cellular genome is methylated. In addition, in non-EBV infected cells it has recently been shown that decitabine can act in combination with chemotherapy to improve its efficacy (Wu et al., 2019). This suggests another avenue of research for combined EBV-associated cancer treatments.

The recent advances that allow for the reversal of DNA methylation and epigenetic silencing of viral gene expression and those that develop specific viral immunotherapies provide promising avenues of research for EBV-associated cancers in the future.

## Author Contributions

The author confirms being the sole contributor of this work and has approved it for publication.

## Conflict of Interest

The author declares that the research was conducted in the absence of any commercial or financial relationships that could be construed as a potential conflict of interest.
